# Underwater image quality assessment method based on color space multi-feature fusion

**DOI:** 10.1038/s41598-023-44179-3

**Published:** 2023-10-06

**Authors:** Tianhai Chen, Xichen Yang, Nengxin Li, Tianshu Wang, Genlin Ji

**Affiliations:** 1 School of Computer and Electronic Information/School of Artificial Intelligence, Nanjing Normal University, Nanjing, 210046 China; 2 School of Artificial Intelligence and Information Technology, Nanjing University of Chinese Medicine, Nanjing, 210023 China

**Keywords:** Computer science, Information technology, Image processing

## Abstract

The complexity and challenging underwater environment leading to degradation in underwater image. Measuring the quality of underwater image is a significant step for the subsequent image processing step. Existing Image Quality Assessment (IQA) methods do not fully consider the characteristics of degradation in underwater images, which limits their performance in underwater image assessment. To address this problem, an Underwater IQA (UIQA) method based on color space multi-feature fusion is proposed to focus on underwater image. The proposed method converts underwater images from RGB color space to CIELab color space, which has a higher correlation to human subjective perception of underwater visual quality. The proposed method extract histogram features, morphological features, and moment statistics from luminance and color components and concatenate the features to obtain fusion features to better quantify the degradation in underwater image quality. After features extraction, support vector regression(SVR) is employed to learn the relationship between fusion features and image quality scores, and gain the quality prediction model. Experimental results on the SAUD dataset and UIED dataset show that our proposed method can perform well in underwater image quality assessment. The performance comparisons on LIVE dataset, TID2013 dataset,LIVEMD dataset,LIVEC dataset and SIQAD dataset demonstrate the applicability of the proposed method.

## Introduction

The underwater world is abundant in resources and images obtained through underwater observation equipment are crucial for exploring the underwater world and exploiting its resources. In practical underwater applications, high-quality underwater images can provide sufficient and accurate information to facilitate the realization of underwater tasks. However, the underwater environment is complex and variable, and factors such as light scattering, absorption, and environmental noise can affect the quality of underwater images, leading to issues such as color shift, low contrast, and low definition^1^.When underwater images are affected by quality degradation, the application value of these images may be reduced^2^, which in turn can impact their performance in practical underwater application tasks. In recent years, numerous effective methods have been proposed for enhancing underwater images^3–9^. However, in the field of underwater image enhancement, there is currently no effective, robust, and widely-accepted method for assessing the quality of underwater images. Therefore, it is of significant research importance to investigate an effective and robust UIQA method. Such an assessment method can help to measure the application value of underwater images and provide a credible and effective reference for the selection of original underwater images and underwater image enhancement methods in practical applications.

There are currently two categories of IQA methods, subjective IQA methods and objective IQA methods^10^. Subjective IQA methods rely on the subjective perception of the observer, which is time-consuming, costly, unstable, and dependent on expert knowledge^11^. As such, subjective methods are only suitable for small-scale image datasets, which hinder the efficient and large-scale quality assessment of underwater images. Therefore, objective IQA methods, which utilize computer-designed methods to automatically and accurately assess image quality, are more suitable for evaluating the quality of underwater images.

IQA methods can be classified according to the amount of reference information required, including Full-Reference Image Quality Assessment (FR-IQA), Reduced Reference Image Quality Assessment (RR-IQA), and No-Reference Image Quality Assessment (NR-IQA)^12,13^. The FR-IQA method requires all the reference information of the original image and compares the difference between the original image and the distorted image to obtain the quality of the distorted image. The RR-IQA method requires partial feature information of the original image as a reference, and the distorted IQA is achieved by calculating the difference between the extracted features of the original image and those of the distorted image. Due to the complexity and uniqueness of the underwater environment, it is often difficult to obtain clear reference images of underwater scenes. Therefore, the NR-IQA method, which requires no original image reference information, is the optimal choice for objective assessment of underwater image quality.

The NR-IQA method does not require the help of a reference image and can access the quality of distorted images based solely on their own features.Moorthy and Bovik^14^ proposed the Blind Image Quality Index (BIQI) based on a two-step framework model of natural scene statistics. The distortions in the image are first identified, and then the statistical features of specific distortion types are extracted. Next, the image authenticity and integrity are evaluated using a controlled pyramid with two scales and six directions, and a corresponding image quality score is calculated for each distortion type. Finally, the weighted average of the quality scores corresponding to different distortions is calculated to obtain the overall IQA results.Li et al.^15^ proposed a no-reference IQA method based on structural degradation to predict the visual quality of multi-distortion images. This method computes gradient-weighted histograms using LBP operators to process gradient images and extract the structural features of images, effectively describing the image quality degradation due to multiple distortions. Liu et al.^16^ proposed a two-step framework IQA model called the Spatial-Spectral Entropy-based Quality Index (SSEQ). This model extracts quality-aware features from three scales of spatial entropy and spectral entropy, and obtains image quality scores by training the model with support vector regression. Gu et al.^17^ proposed the Blind Image Quality Measure of Enhanced Images (BIQME), which analyzes the contrast, sharpness, and brightness of images based on 17 features to assess image quality.Mittal et al.^18^ proposed a spatial domain-based no-reference quality assessment method Blind/Referenceless Image Spatial Quality Evaluator (BRISQUE), which uses the statistical properties of the empirical distribution of local brightness normalization to quantify the image quality based on the morphological features of the image. Based on this approach, Mittal et al.^19^ introduced an unsupervised Natural Image Quality Evaluator (NIQE) use the statistical features of natural scenes. The NIQE method uses a multivariate Gaussian (MVG) model to fit the image. Zhang et al.^20^ extended Integrated Local NIQE(ILNIQE) based on NIQE, which adds more image-related features and calculates the distance between the image patches and the Gaussian model, finally integrates the image patch quality scores to get the overall image quality score. In recent years, deep learning-based methods and techniques have been widely employed in the task of IQA , Kang et al.^21^ proposed a convolutional neural network-based IQA method Convolutional Neural Networks for no-reference Image Quality Assessment (CNNIQA), which mainly contains one convolutional layer and two fully connected layers, integrating feature extraction and assessment model building into one network. Zhang et al.^22^ introduced a Deep Bilinear Convolutional Neural Network (DBCNN) for NR-IQA, which is applicable to both synthetic and real distorted images. The DBCNN consists of two linear convolutional neural networks, each dedicated to a distorted scene. Features from both CNNs are bilinearly merged for the final quality prediction. Su et al.^23^ presented an adaptive hyper network architecture that first extracts image semantics and establishes perceptual rules through hypernetwork adaptation, which is adopted by the quality prediction network to give image quality. You et al.^24^ first applied Transformer to the field of IQA and proposed Transformer for Image Quality assessment (TRIQ) method. TRIQ uses an adaptive encoder to process images of different resolutions, and the feature maps extracted by the convolutional neural network are used as the input to the Transformer encoder, and then the image quality is sensed by the MLP Head. Yang et al.^25^ extended Multi-dimension Attention Network for No-Reference Image Quality Assessment (MANIQA) based on a multi-dimensional attention network. The method extracts features by Vision Transformer (ViT), applies attention mechanisms in channel and spatial dimensions using transfer attention blocks and scaling transformation blocks, using a two-branch structure of patch weighted quality prediction to predict the final score based on the weight of each patch quality score.

In recent years, as the field of underwater image processing has continued to advance, a number of UIQA methods have been proposed. Yang et al.^26^ proposed the Underwater Color Image Quality Evaluation Metric (UCIQE), which extracts underwater image features that align more closely with human perception in the CIElab color space. The saturation, chromaticity, and contrast of underwater images are used as measurement components, which are subsequently combined linearly to develop a quality assessment method capable of measuring color shift, blurring, and low contrast of underwater images. Panetta et al.^27^ proposed an Underwater Image Quality Measure (UIQM) method based on the Human Visual System (HVS). The UIQM method considers various factors that impact the quality of underwater images by utilizing the Underwater Image Colorfulness Measure (UICM), Underwater Image Sharpness Measure (UISM), and Underwater Image Contrast Measure (UIConM). The method combines these three measurement components linearly to comprehensively characterize the quality of underwater images. In addition to the aforementioned methods, Yang et al.^28^ proposed a no-reference frequency domain-based UIQA method called FDUM. The FDUM method combines the spatial and frequency domains in the color metric, refines the contrast metric using a dark channel a priori method, and uses multiple linear regression to obtain weighting coefficients for the linear combination of the metric values in order to assess the quality of underwater images. On the other hand, Zheng et al.^29^, on the other hand, proposed an Underwater Image Fidelity (UIF) metric. The metric relies on statistical features of underwater images in the CIELab space, suggesting naturalness, sharpness, and structure indices. Saliency-based spatial pooling is used to determine the final quality score of the image. While RGB-based IQA methods have demonstrated advantages in assessing the quality of natural scene images^30,31^, they do not perform well for underwater scenes due to the high correlation and poor uniformity of their components, which deviate significantly from human visual perception^29^. Current methods for assessing the quality of underwater images inadequately use color space, which leads to poor feature extraction and an inaccurate representation of the relationship between underwater image quality and subjective perception. Presently, these methods primarily consider chromaticity, contrast, sharpness, saturation, and similar features, with relatively limited feature selection. As a result, they fail to recognize that the degradation of underwater image quality can be caused by a combination of several factors.

To address the issues with existing methods, this paper proposes a No-Reference Underwater Image Quality Assessment based on Multi-feature Fusion in Color Space (NMFC). The NMFC method utilizes a color space transformation method to extract luminance histogram, Local Binary Pattern (LBP), moment statistics, and morphological features in the CIELab color space to represent underwater image information through feature fusion. Support Vector Regression (SVR) is subsequently used to learn the relationship between the fused features and underwater image quality, enabling the establishment of a model and the realization of accurate UIQA . Experimental results obtained using the SAUD^32^ and UIED^29^ underwater image quality assessment datasets demonstrate that the proposed method effectively and accurately assesses underwater image quality and exhibits high consistency with human subjective perception. The generality of the method is further demonstrated using several traditional image datasets, including LIVE^33^, TID2013^34^, LIVEMD^35^, LIVEC^36^ and SIQAD^37^.

## Methods

**Figure 1:**
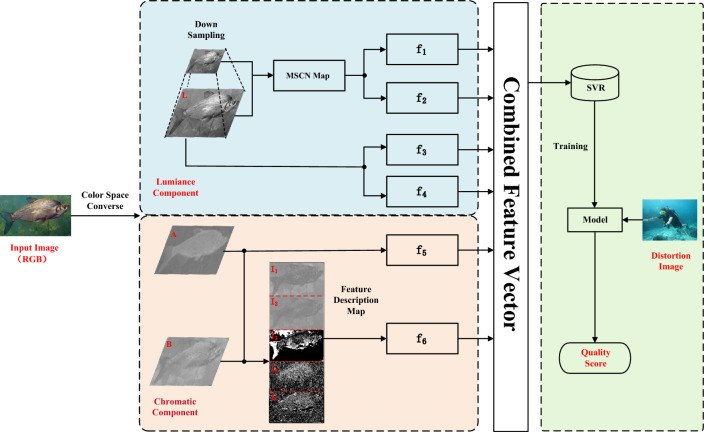
The detailed procedure of the proposed method.

The proposed method is illustrated in Fig. 1,begins by converting underwater images from RGB to CIELab color space through color space conversion. In this color space, six quantified underwater image distortion features (as detailed in Table 1) are extracted and fused with multiple features to form a feature vector. Finally, SVR is utilized to learn the mapping relationship between the feature vectors and the quality of underwater images, resulting in the establishment of a model for UIQA.

**Table 1:** Details of features extracted by this method.

Feature ID	Feature description
$f_{1}$	LBP histogram feature
$f_{2}$	Luminance component morphological feature
$f_{3}$	Luminance component moment statistic feature
$f_{4}$	Luminance histogram feature
$f_{5}$	Chromatic component moment statistic feature
$f_{6}$	Chromatic component morphological feature

### Color space conversion

The CIELab color space^38^ is specifically designed to match human color perception and achieve perceptual uniformity. Consequently, in order to more effectively extract features, the proposed method converted the RGB underwater images to the CIELab color space. Specifically, the proposed method first transformed the images from the RGB color space to the XYZ color space, before subsequently converting them to the CIELab color space. Eq. (1) was used to accomplish this conversion, as detailed below.

1 $$\begin{aligned} \left[ \begin{array}{llll}X\\ Y\\ Z\end{array}\right] =\left[ \begin{array}{llll} 0.412453&{}0.357580&{}0.180423\\ 0.212671&{}0.715160&{}0.072169\\ 0.019334&{}0.119193&{}0.950227\\ \end{array}\right] \left[ \begin{array}{llll}R\\ G\\ B \\ \end{array}\right] \end{aligned}$$

The equation for converting XYZ color space to CIELab color space is as follows:

2 $$\begin{aligned} \begin{array}{l} \left\{ \begin{matrix} L^*=116f(Y/Y_{n})-16 \\ a^*=500[f(X/X_{n})-f(Y/Y_{n})] \\ b^*=200[f(Y/Y_{n})-f(Z/Z_{n})]\end{matrix}\right. \end{array}\ \end{aligned}$$

3 $$\begin{aligned} f(t)=f(x)=\left\{ \begin{array}{ll} t^{1 / 3}, &{} t>\left( \frac{6}{29}\right) ^{3} \\ \frac{1}{3}\left( \frac{29}{6}\right) ^{2} t+\frac{4}{29}, &{} \text{ otherwise } \end{array}\right. \end{aligned}$$

Where, $X_{n},Y_{n},Z_{n}$ is the CIE XYZ trichromatic stimulus value of the reference white point value [0.9504, 1.0000, 1.0888], simulating noon sunlight with the associated color temperature of 6504 K.

**Figure 2:**
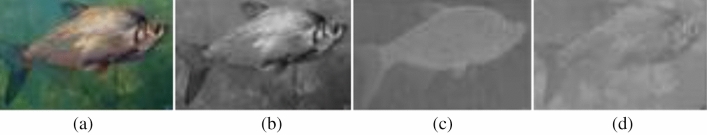
Examples of underwater images and the corresponding component images. (**a**) Raw image, (**b**) L-component image, (**c**) A-component image, (**d**) B-component image.

Figure 2b–d depict the image data corresponding to the L-component, A-component, and B-component of the original image Fig. 2a subsequent to the color space conversion.

### L component feature extraction

Underwater images are known to be impacted by the absorption and scattering of light by water, often resulting in insufficient brightness, low visibility, and low contrast, thereby leading to a degradation of underwater image quality. It is widely acknowledged that the luminance channel L component plays a crucial role in this regard. Therefore, there is a need to extract features that can effectively characterize changes in underwater image quality, with a particular emphasis on the L component.

#### L component luminance histograms

The luminance histogram features can be calculated by Eq. (4), the process is: (1) divide the image luminance values into *k* bins, (2) traverse the image pixels and count the number of pixels in each bin, (3) divide the number of pixels in each bin by the total number of pixels in the image to get the probability of each bin, thus the luminance histogram features are obtained.

This method is based on the luminance component map by Eq. (4) to obtain the corresponding histogram features of the underwater image as histogram features $f_{4}$.

4 $$\begin{aligned} H(k)=\frac{1}{MN}\sum _{i=1}^{M} \sum _{j=1}^{N}\delta (p_{ij} = k) \end{aligned}$$

where, $i\in \left\{ 1,2,...,M \right\} ,j\in \left\{ 1,2,...,N \right\}$,*M*, *N* are the height and width of image $I,p_{ij}$ is the pixel value,$\delta$ is the indicating function, which is 1 when$p_{ij} = k$ and 0 otherwise.

#### L component morphological parameters

Previous studies have suggested that performing nonlinear operations on images can help eliminate the correlation between pixels in the image^39^. Hence, to extract features from the underwater images I , this study utilizes the Mean Subtracted Contrast Normalized (MSCN) technique (as depicted in Eq. 5) and extracts statistical features based on the MSCN coefficients^16^. The extraction process is performed as follows.

*I* is an underwater image of size M*N, which has MSCN coefficient :

5 $$\begin{aligned} {\hat{I}}=\frac{I(i,j)-\mu (i,j)}{\sigma (i,j)+C} \end{aligned}$$

where:$\mu (i,j)$ is the result after Gaussian filtering, and$\sigma (i,j)$ is the standard deviation . C is the constant 1 that prevents the denominator from being 0. The variables $\mu (i,j)$ and $\sigma (i,j)$ are defined as follows:

6 $$\begin{aligned} \mu (i,j)=\sum _{k=-K}^{K}\sum _{l=L}^{L}w_{k,l}I(i+k,j+l) \end{aligned}$$

7 $$\begin{aligned} \sigma (i,j)=\sqrt{\sum _{k=-K}^{K}\sum _{l=L}^{L}w_{k,l}[I(i+k,j+l)-\mu (i,j)]^2} \end{aligned}$$

8 $$\begin{aligned} w=\left\{ w_{k,l}=-K,...,K,l=-L,...,L\right\} \end{aligned}$$

where *w* is the two-dimensional cyclic symmetric Gaussian weight function, and($K=L=3$) .

The statistical characteristics are obtained by fitting the MSCN coefficients using the Asymmetric Generalized Gaussian Distribution (AGGD) model. The AGGD model is shown in Eq. (9)

9 $$\begin{aligned} f\left( x; \alpha , \sigma _{l}^{2}, \sigma _{r}^{2}\right) =\left\{ \begin{array}{l}\frac{\alpha }{\left( \beta _{l}+\beta _{r}\right) \Gamma \left( \frac{1}{\alpha }\right) } \exp \left( -\left( \frac{-x}{\beta _{l}}\right) ^{\alpha }\right) , \forall x \le 0 \\ \frac{\alpha }{\left( \beta _{l}+\beta _{r}\right) \Gamma \left( \frac{1}{\alpha }\right) } \exp \left( -\left( \frac{-x}{\beta _{r}}\right) ^{\alpha }\right) , \forall x \ge 0\end{array}\right. \end{aligned}$$

where,

10 $$\begin{aligned} \beta _{l}=\sigma _{l}\sqrt{\Gamma (1/\alpha )/\Gamma (3/\alpha )} \end{aligned}$$

11 $$\begin{aligned} \beta _{r}=\sigma _{r}\sqrt{\Gamma (1/\alpha )/\Gamma (3/\alpha )} \end{aligned}$$

12 $$\begin{aligned} \eta =(\beta _{r}-\beta _{l} )\Gamma (\frac{2}{\alpha } )/\Gamma (\frac{1}{\alpha })\end{aligned}$$

13 $$\begin{aligned} \Gamma (x)=\int _{0}^{\infty } t^{x-1}e^{-t}dt, x>0 \end{aligned}$$

$\Gamma (x)$ is the gamma function;t is any one of the variables taking the value $[0,+\infty ]$.

The MSCN coefficients were obtained by AGGD fitting using the moment matching method^40^ for the shape parameters $\alpha$,the left and right scale parameters $\sigma _{l}^2,\sigma _{r}^2$, the mean value $\eta$ .The skewness S as well as the kurtosis K^41^ of the MSCN image are calculated Eqs. (14) and (15).Shape parameters $\alpha$, left and right scale parameters $\sigma _{l}^2,\sigma _{r}^2$, mean $\eta$ skew S and kurtosis K are taken as morphological features$F_S^{\forall }=(\alpha ^\forall ,\eta ^\forall ,\sigma _{l} ^\forall ,\sigma _{r}^\forall ,S^\forall ,K^\forall )$ and $\forall$ denote any one image.

14 $$\begin{aligned} S= & {} \frac{\frac{1}{n} \sum _{i=1}^{n}(x_{i}-{\bar{x}})^3}{(\frac{1}{n} {\textstyle \sum _{i=1}^{n}(x_{i}-{\bar{x}})^2 } )^{\frac{3}{2} }} \end{aligned}$$

15 $$\begin{aligned} K= & {} \frac{\frac{1}{n} \sum _{i=1}^{n}(x_{i}-{\bar{x}})^4}{(\frac{1}{n} {\textstyle \sum _{i=1}^{n}(x_{i}-{\bar{x}})^2 } )^{2 }} \end{aligned}$$

Downsampling is applied to the luminance component map, and for the image $I(M*N)$, the proposed method downsample it to obtain a downsampled map ${\tilde{I}}$ with a resolution of$(\frac{M}{2})*(\frac{N}{2})$.The morphological features of I and the downsampled map${\tilde{I}}$as the features of $f=[F_S^I,F_S^{{\tilde{I}}}]$.

#### L component moment statistics

The moment statistics^42^ of the luminance component maps, including the first-order moment mean $\mu$, second-order moment variance $\sigma$, third-order moment skewness *s*, and fourth-order moment kurtosis *k*, were calculated using equations Eqs. (16–19).

16 $$\begin{aligned} \mu _{i}= & {} \frac{1}{N}\sum _{j=1}^{N}p_{ij} \end{aligned}$$

17 $$\begin{aligned} \sigma _{i}= & {} (\frac{1}{N}\sum _{j=1}^{N}(p_{ij}-\mu _{i})^2)^{\frac{1}{2} } \end{aligned}$$

18 $$\begin{aligned} s _{i}= & {} (\frac{1}{N}\sum _{j=1}^{N}(p_{ij}-\mu _{i})^3)^{\frac{1}{3} } \end{aligned}$$

19 $$\begin{aligned} k _{i}= & {} (\frac{1}{N}\sum _{j=1}^{N}(p_{ij}-\mu _{i})^4)^{\frac{1}{4} } \end{aligned}$$

where $p_{ij}$ represents the first pixel of the *j* pixel of the *i* component of the image and N represents the number of pixels in the image.

The four moment statistics are used as features $f_{3}$ ,which means $f_{3}=[\mu _{L},\sigma _{L},s_{L},k_{L}]$ .

#### L component LBP histograms

The proposed method use the rotationally invariant uniform LBP operator^43^ for the image *I* and its downsampled map ${\tilde{I}}$ .The rotation-invariant uniform LBP model is defined as:

20 $$\begin{aligned}{} & {} L B P_{P, R}^{\text{ riu2 } }\left( p_{i j}\right) =\left\{ \begin{array}{c} \sum _{n=0}^{P-1} f\left( p_{n}-p_{i j}\right) , U_{i j} \le 2 \\ P+1, \text{ otherwise } \end{array}\right. \end{aligned}$$

21 $$\begin{aligned} f(x)= & {} \left\{ \begin{array}{c}1,x\ge 0 \\ 0, x< 0\end{array}\right. \end{aligned}$$

Where $p_{ij}$ represents the center point (*i*, *j*) pixel value. $p_{n}$ represents the nth pixel value of the neighborhood, *P* is the number of neighborhoods, *R* is the radius of the neighborhood, and $U_{ij}$ is the pixel consistency parameter of the pixel point (*i*, *j*) the pixel consistency parameter of the neighboring pixel points, defined as:

22 $$\begin{aligned} \begin{aligned}{}&U_{ij} =|f(p_{P-1},p_{ij})-f(p_{0},p_{ij})|\\&+\sum _{p=0}^{P-1} |f(m_{p},m_{ij})-f(m_{p-1},m_{ij})| \end{aligned} \end{aligned}$$

After processing the two maps using rotation-invariant uniform LBP, the LBP histogram is built based on the two LBP mapping maps by the Eqs. (23) and (24):

23 $$\begin{aligned} H(k)= & {} \frac{1}{MN}\sum _{i=1}^{M} \sum _{j=1}^{N}\delta (LBP_{P,R}^{riu2},k) \end{aligned}$$

24 $$\begin{aligned} \delta (x)= & {} \left\{ \begin{array}{c}1,x=y \\ 0, otherwise\end{array}\right. \end{aligned}$$

The obtained LBP histogram feature can effectively perceive the underwater image quality, so it is used as the luminance component feature $f_{1}$ .

### AB component feature extraction

In order to quantify the degradation of underwater image quality resulting from color changes, our proposed method extracts features from the chromatic channels.

#### Chromatic feature maps

Inspired by the NUIQ method^32^build chromatic descriptor maps based on the two chromatic components($O_{1}$ and $O_{2}$ ) to extract the chromatic component features of underwater images.The proposed method constructs five color feature maps, AB difference map, saturation map, AB angle map and AB derivation angle, based on A and B chromatic components,which can represent the color perception information of underwater images from different perspectives and effectively describe the features of chromatic channels in underwater images. The construction process of the chromatic feature maps is defined as Eq. (25):

25 $$\begin{aligned} \begin{array}{c} a_{1}=|A-B| \quad a_{2}=\sqrt{A^{2}+B^{2}} \quad a_{3}=\arctan \left( \frac{A}{B+c}\right) \\ a_{4}=\arctan \left( \frac{\nabla _{x} A}{\nabla _{x} B+c}\right) \quad a_{5}=\arctan \left( \frac{\nabla _{y} A}{\nabla _{y} B+c}\right) \end{array} \end{aligned}$$

Where $\nabla _{x},\nabla _{y}$ is the gradient along the horizontal and vertical directions. *c* is a small constant.

**Figure 3:**
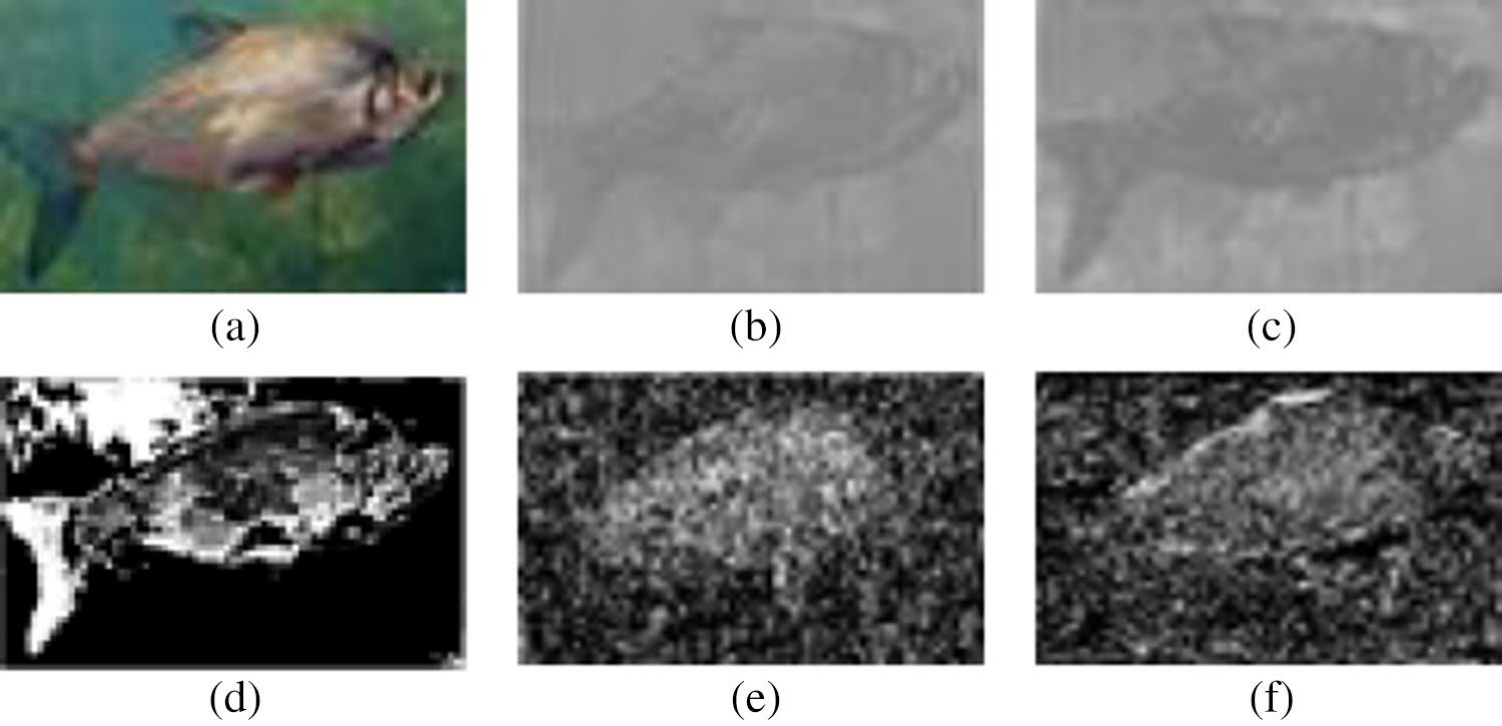
Illustration of chromatic feature maps. (**a**) Pristine underwater image, (**b–f**) are five chromatic feature maps of (**a**).

The five feature maps feature maps $a_{1}-a_{5}$ are illustrated in Fig. 3

**Figure 4:**
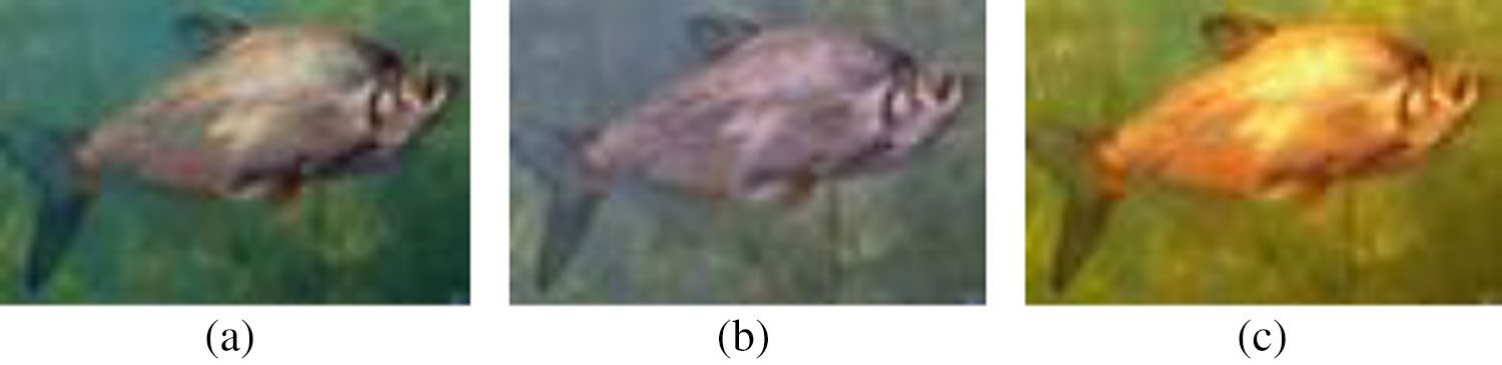
Examples of underwater images. (**a**) I1, (**b**) I2, (**c**) I3.

**Figure 5:**
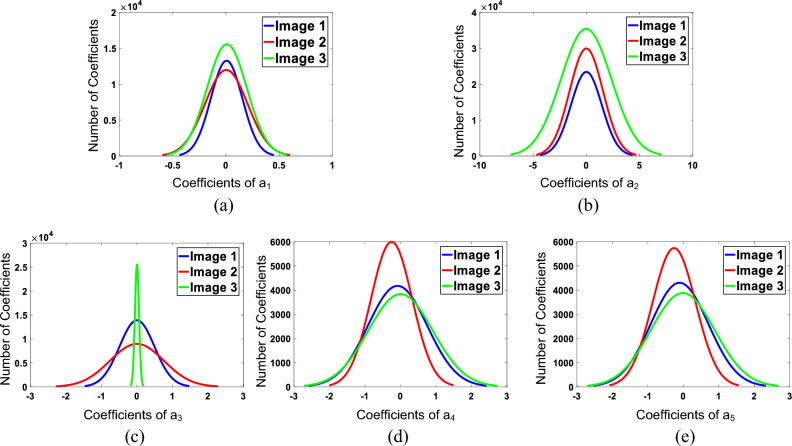
Fitting curves of the chromatic feature maps for the underwater images shown in Fig.4.

To confirm the effectiveness of our proposed chromatic feature maps, three underwater images with varying image quality (as shown in Fig. 4) were selected. The corresponding five chromatic feature maps were fitted with a normal distribution function, and the resulting curves are shown in Fig. 5.

#### AB component morphological parameters

In Fig. 5, the subfigure displays fitted curves of color feature maps corresponding to three different quality levels of underwater images. Each fitted curve represents a distinct shape, highlighting the variations in the color component morphological features.This confirms that the five chromatic feature maps constructed in this study successfully reflect the variations in underwater image quality. Consequently, it is reasonable to utilize the morphological parameters of the fitted curves of these chromatic feature maps as morphological features for the analysis of underwater image quality.

Based on the five chromatic feature maps AGGD fitting was applied Eq. (9) to obtain the shape parameter $\alpha$, the left and right scale parameters $\sigma _{l}^2,\sigma _{r}^2$, and the mean value of the distribution $\eta$. For the five chromatic feature maps skewness S and kurtosis K were calculated according to Eqs. 14 and 15. Combining the five feature maps corresponding parameters as the feature $f_{6}$, that is $f_{6}=[F_{S}^{a_{1}},F_{S}^{a_{2}},F_{S}^{a_{3}},F_{S}^{a_{4}},F_{S}^{a_{5}}]$.

#### AB component moment statistics

Similar to the luminance channel, this method also performs the calculation of moment statistics in the chromatic channel.

This method will use Eq. (16–19) to calculate the first-order moment mean $\mu$,second-order moment variance $\sigma$, third-order moment skewness *s* and fourth-order moment kurtosis *k* on the A-component map and B-component map of the chromatic channel to obtain a moment vector as the chromatic channel moment statistic $f_{5}$, then $f_{5}=[\mu _{A},\sigma _{A},s_{A},k_{A},\mu _{B},\sigma _{B},s_{B},k_{B}]$

### Building image quality assessment model

Feature fusion can realize the complementary features of chromatic and luminance components of underwater images, and describe the quality change of images more effectively. After the two types of features are extracted, feature fusion will be performed. We employ a feature fusion strategy that concatenates multiple features $f_{1}-f_{6}$ to obtain fused features $F=[f_{1},f_{2},f_{3},f_{4},f_{5},f_{6}]$. However, the process of building an IQA model remains to be addressed. Considering the impressive generalization ability of SVR along with its widespread use in numerous methods for IQA^14,16,17^, it is deemed suitable for accurately establishing relationships between image quality-related features and image scores. Therefore, for the purposes of this study, the LIBSVM toolkit^44^ was utilized to implement SVR with a Radial Basis Function (RBF) kernel. The mapping between fused features and subjective quality scores of underwater images was established using SVR in order to gain an UIQA model. In evaluating the quality of underwater images, features are extracted from the images to be measured according to the method outlined in this paper. These features are then fused and input into the UIQA model to obtain the quality score.

## Experimental results

### Dataset and assessment metric

In order to analyze the performance of the proposed method, this paper conducted comparative experiments on publicly available image datasets: SAUD^32^, UIED^29^, LIVE^33^,LIVEMD^35^,LIVEC^36^,TID2013^34^, and SIQAD^37^. Each dataset contains subjective IQA results for each image, including mean opinion score (MOS), difference mean opinion score (DOMS), or Bradley–Terry score (B–T score)^45^.

**Figure 6:**
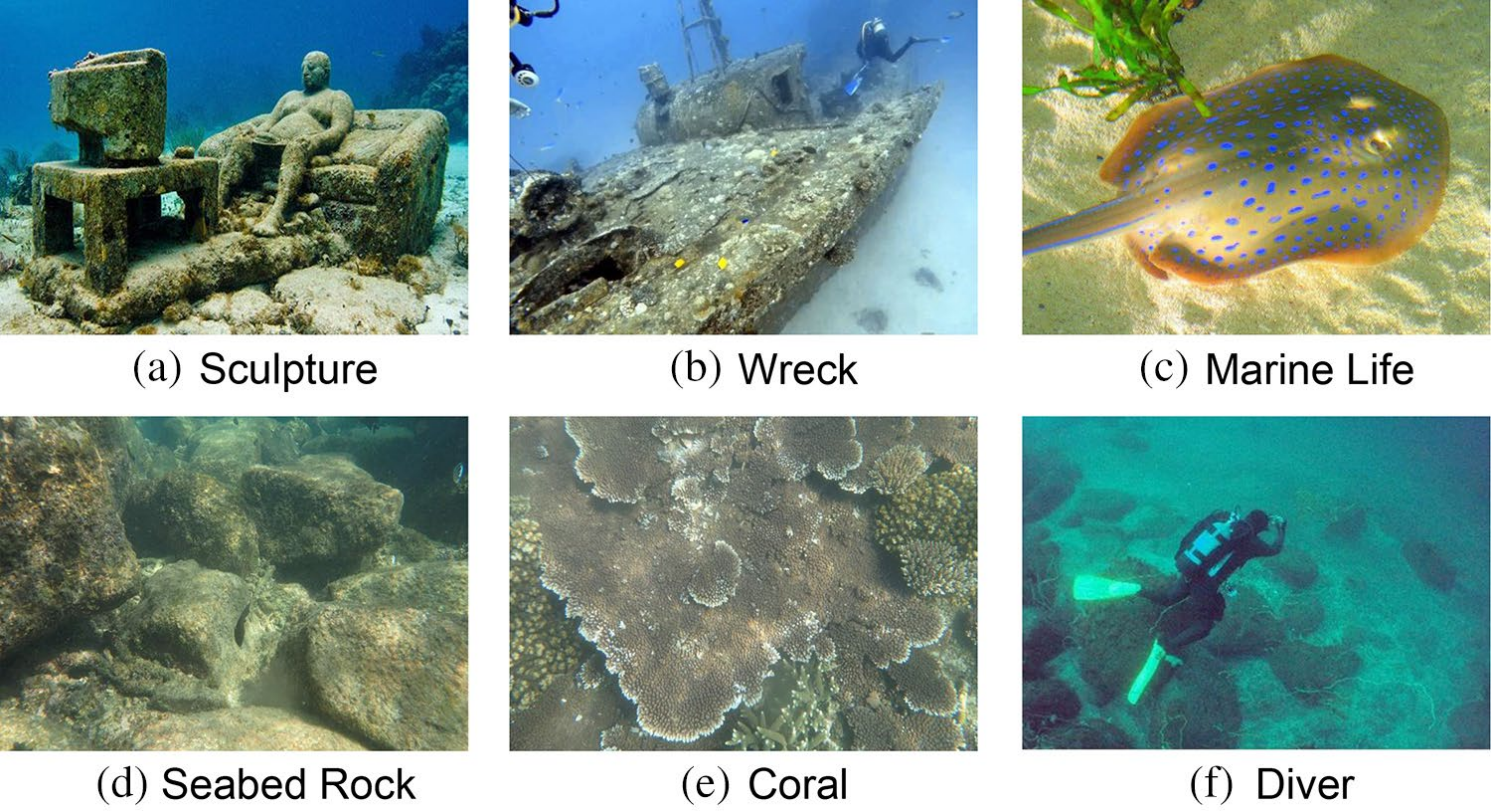
Typical underwater images in UIED database.

The UIED dataset^29^ includes 100 images of real underwater scenes,including 16 coral images,26 marine life images, 14 seabed rock images, 12 sculpture images, 10 wreck images and 22 diver images.Typical images in the database are shown in Fig. 6, with resolutions ranging from 183 $\times$ 275 to 1350 $\times$ 1800. 10 representative underwater image enhancement algorithms are used to process the original images, and the resulting dataset was comprised of 1000 images. The SAUD database^32^ is similar to the UIED database, as it is also an underwater image quality evaluation dataset,also containing 100 images of real underwater scenes. However, different enhancement methods were selected for processing the underwater images in this dataset. The LIVE^33^ database contains 29 distortion-free high-resolution images as reference images and 779 distorted images corresponding to the reference images. These images have five distortion types, which are (1) 175 JPEG2000 Compression(JP2K) distorted images; (2) 169 JPEG Compression (JPEG) distorted images; (3) 145 Gaussian Blur (GB) distorted images; (4) 145 White Gaussian Noise (WN) images; and (5) 145 Fast Fading error (FF) distorted images.LIVE image database provides a DMOS for each image in the range [0, 100], with higher scores representing poorer image quality. The TID2013^34^ database contains 25 reference images and their corresponding distortion images of 24 distortion types, each containing five distortion levels, for a total of 3000 images, giving MOS values in the range [0, 9] to indicate perceptual quality. The LIVEMD^35^ database is a mixed distortion image database containing two types of multiple distortion, which are blur distortion mixed with JPEG compression distortion and Gaussian blur distortion mixed with Gaussian white noise.Fifteen reference images were used to generate 450 distorted images in both multiple distortion scenarios, with 225 images of both types, and each image was given a range [0, 100] of DMOS only as a subjective score. LIVE Challenge^36^ is an authentic IQA database containing 1162 images. Each image is captured by a diverse photographer using distinct camera equipment ,encompassing a wide range of real-world scenes, and these images suffer from complex reality distortion. The MOS value corresponding to each image is obtained through an online crowdsourcing platform in the range [0, 100]. SIQAD^37^ is a commonly used screenshot image database that contains 20 reference images and their corresponding 980 distorted images with seven distortion levels for each distortion type. The database contains seven distortion types, which are GN, GB, Motion Blurring (MB), Contrast Change (CC), JPEG, JP2K and Layer Segmentation based Compression (LSC).SIQAD gives the DMOS value for each image in the range [0, 100].The specific information for each dataset is presented in Table 2.

Experimental analysis conducted on the SAUD and UIED image datasets can effectively verify the effectiveness of the proposed method for UIQA. The experiments carried out on LIVE, LIVMD, LIVEC, TID2013, and SIQAD demonstrate the applicability of the proposed method for different types of image data.

**Table 2:** Detailed information of benchmark datasets.

Dataset	Ref Images	Distortion images	Distortion types	Subjective scores
SAUD	100	1000	10	B–T score
UIED	100	1000	10	MOS
LIVE	29	779	5	DMOS
SIQAD	20	980	7	MOS
TID2013	25	3000	24	MOS
LIVEMD	15	450	2	DMOS
LIVEC	–	1162	–	MOS

To evaluate the performance of various objective IQA methods, it is necessary to compare the subjective assessment scores of images with the IQA results obtained by these methods. If an objective IQA method demonstrates high agreement with the subjective image assessment scores, it indicates that the corresponding method is more consistent with human subjective perception and has better performance. This paper utilize three widely adopted metrics, including the Spearman rank-order correlation coefficient (SROCC), Kendall rank-order correlation coefficient (KROCC), and Pearson linear correlation coefficient (PLCC), to measure the performance of IQA methods. The SROCC and KROCC values measure the monotonicity between the method’s assessment results and the subjective assessment scores, while the PLCC value measures the accuracy of the method for IQA. The closer the absolute values of these three metrics are to 1, the higher the agreement between the assessment results of the corresponding objective quality assessment method and the subjective assessment scores, indicating better performance by the method.

### Performance analysis of the method

To evaluate the effectiveness of the proposed NMFC method, this study conducted comparisons between our method and mainstream IQA methods on two datasets, including the SAUD dataset and the UIED dataset. The proposed method selected seven existing NR-IQA methods for comparison, including BIQI^14^, SSEQ^16^, BRISQUE^18^, NIQE^19^, ILNIQE^20^, BIQME^17^, NUIQ^32^, and classical UIQA methods,including UIQM^26^, UCIQE^27^, FDUM^28^,UIF^29^. Among these methods, the NIQE and ILNIQE approaches do not require subjective scoring and are thus regarded as fully blind IQA methods, which therefore do not require retraining. The remaining methods were retrained using the SAUD and UIED datasets. In order to ensure the fairness of the experiments, this paper utilized the authors’ published source code and trained the score assessment models using SVR in all experiments.

For image dataset division, the proposed method randomly split the images into a training set and a test set, with 80$\%$ and 20$\%$ of the images respectively. Additionally, the two parts were ensured to contain no duplicate images. The score assessment model was then trained using all the features extracted from the images in the training set, along with their corresponding subjective assessment scores. Once the model was obtained, it was used to evaluate the images in the test set and calculate the appropriate assessment metrics. To ensure the accuracy and reliability of the experimental results, proposed method conducted multiple iterations of the experiment. Specifically, the process was repeated 50 times for each method, with 50 rounds of training and testing carried out for every iteration. After 50 rounds of testing, we calculated the average value of each assessment metric, which was then taken as the overall assessment result of the corresponding IQA method.

Table 3 gives the overall performance comparison results of this paper method with other methods on SAUD and UIED datasets, and the values of the best and second performance corresponding to different assessment metrics on each dataset are bolded. According to the experimental results in Table 3, we can get the following conclusions. First, the method proposed in this paper outperforms other IQA methods on the underwater image dataset. SROCC, KROCC, PLCC assessment index values corresponding to the overall assessment results of this method on the SAUD and UIED image datasets reach the optimum; NUIQ method achieves sub-optimal SROCC, KROCC, PLCC values on the SAUD and UIED dataset. Compared with the suboptimal method, the values of SROCC, KROCC, PLCC on the SAUD dataset are higher than the suboptimal method by 0.0463, 0.0443 and 0.0397; the values of SROCC, KROCC, PLCC on the UIED dataset are higher than the suboptimal method by 0.0215, 0.0188 and 0.0055 or so. Secondly,the UCIQE, UIQM, and FDUM algorithms exhibit subpar performance on the SAUD and UIED databases, as evidenced by their low SROCC, KROCC, and PLCC values. It is worth noting that the UIF method underperforms specifically on the SAUD database; however, it ranks third in terms of SROCC (0.6117), KROCC (0.4428), and PLCC (0.631) values for the UIED dataset. This discrepancy can be attributed to these methods’ reliance on combining measurement component scores with weights to derive quality scores for underwater images.As described in the related work above, limitation of such approaches lies in their dependence on weight distributions constrained by specific databases, where coefficients differ across different datasets.This also one of the factors that motivated the design of our proposed method. Lastly, the performance of some methods is poor, and the assessment index values of SROCC and PLCC are less than 0.5, which is a big gap with the methods in this paper. According to the above conclusions, it can be seen that the IQA method proposed in this paper has certain advantages compared with other IQA methods on different underwater image datasets, and the consistency with human subjective perception is higher than other methods, which can efficiently and accurately evaluate the quality of underwater images.

**Table 3:** Performance comparison of the proposed method on two underwater image datasets.

IQA method	SAUD			UIED		
	SROCC	KROCC	PLCC	SROCC	KROCC	PLCC
BRISQUE	0.4934	0.3481	0.5180	0.3964	0.2741	0.4306
BIQI	0.4057	0.2820	0.4269	0.4438	0.3089	0.5196
NIQE	0.0626	0.0425	0.1814	0.2528	0.1702	0.2198
ILNIQE	0.2844	0.1929	0.2825	0.4196	0.2882	0.4066
SSEQ	0.5475	0.4249	0.5683	0.5161	0.3642	0.5577
BIQME	0.7271	0.5924	0.7483	0.5194	0.3681	0.6183
UIQM	0.0271	0.0179	0.0581	0.0839	0.0562	0.0560
UCIQE	0.2002	0.1312	0.4351	0.0591	0.0438	0.2465
UIF	0.1182	0.0709	0.2021	0.6117	0.4428	0.6316
FDUM	0.1469	0.0965	0.2819	0.0865	0.0620	0.1011
NUIQ	0.7891	0.6024	0.8004	0.6136	0.4459	0.6761
NMFC	**0.8354**	**0.6467**	**0.8401**	**0.6351**	**0.4647**	**0.6816**

#### Intuitive comparison

**Figure 7:**
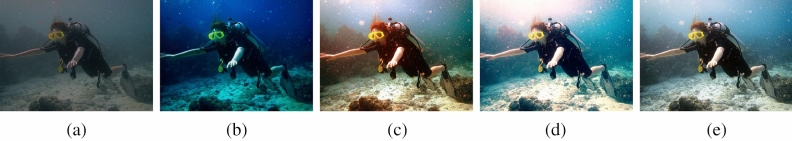
Figures (**a–e**) represent a collection of underwater images, each accompanied by a subjective rating. The images have been ranked in ascending order based on their respective subjective scores.

**Figure 8:**
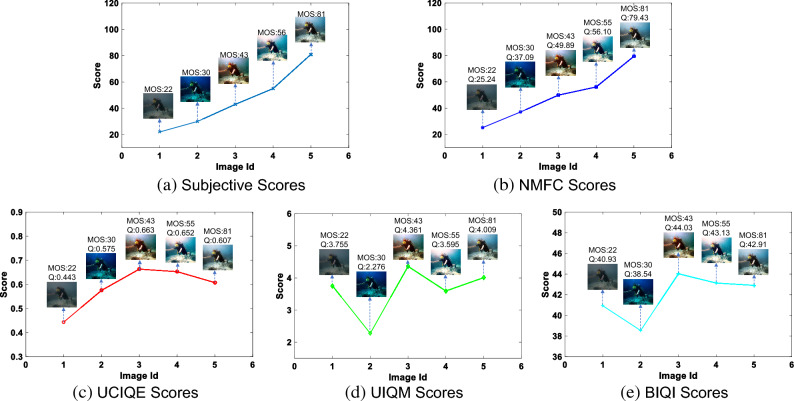
Prediction quality values of different methods on underwater image (Fig. 7). Q is the predicted quality score.

To facilitate a more intuitive comparison between the proposed method and other methods, this research paper presents a series of underwater images with varying subjective quality scores in Fig. 7. These images are arranged in ascending order from left to right based on their respective scores. Various quality assessment methods are employed to evaluate these images, and the resulting subjective quality scores, NMFC values, UCIQE values, UIQM values, and BIQI values are depicted in Fig. 8. Additionally, line charts are utilized to emphasize the effectiveness of the proposed method in comparison to other IQA methods.

From the figure, it can be observed that the traditional underwater image quality evaluation methods, UCIQE and UIQM, primarily focus on the color components of the image. As a result, images with rich colors (such as Fig. 7c) often receive higher scores, leading to the highest evaluation for images with medium quality. On the other hand, the traditional air IQA method, BIQI, is designed for assessing image quality in natural scenes and does not take into account the factors that affect image quality in the underwater environment. Consequently, its performance is limited, and it is unable to differentiate the quality of underwater images. In contrast, the proposed NMFC method captures features that can effectively describe the degradation of underwater image quality, considering both the luminance and chromatic components. The scores obtained through this method demonstrate better alignment with subjective quality scores, thus achieving superior performance.

### Ablation experiments

To validate the chosen CIELab color space for evaluating underwater image quality in this paper, ablation experiments were conducted on the SAUD and UIED databases using the CIElab color space, opponent color space, and YCbCr color space. The experimental results are presented in Table 4.

Based on the results presented in Table 4, the following conclusions can be drawn. Firstly, on the SAUD database, the method utilizing the CIELab color space outperforms the sub-optimal method employing the Opponent color space, with higher values of SROCC, KROCC, and PLCC evaluation indices by 0.0051, 0.0037, and 0.0014, respectively. Secondly, on the UIED database, the method utilizing the CIELab color space achieves the highest SROCC and KROCC values. However, the PLCC values is slightly lower compared to the method using the Opponent color space, with only a marginal difference of 0.0125 in the PLCC evaluation metric.Moreover, the utilization of the YCbCr color space yields unsatisfactory outcomes on both datasets. The SROCC, KROCC, and PLCC evaluation metrics exhibit lower values in comparison to the other two color spaces. This disparity suggests that the YCbCr color space inadequately captures color information, resulting in an inability to depict image intricacies and color variations. Consequently, the evaluation of underwater image quality suffers from diminished performance. In summary, the findings highlight the superior efficacy of employing the CIELab color space for underwater image quality assessment when contrasted with the alternative color spaces.

**Table 4:** Performance comparison of the proposed method on different color space.

Color Space	SAUD			UIED		
	SROCC	KROCC	PLCC	SROCC	KROCC	PLCC
Opponent	0.8303	0.6440	0.8387	0.6346	0.4635	**0.6941**
YCbCr	0.8239	0.6367	0.8328	0.6286	0.4598	0.6793
CIELab	**0.8354**	**0.6467**	**0.8401**	**0.6351**	**0.4647**	0.6816

The best values are in bold.

Furthermore, a series of ablation experiments were conducted on the SAUD dataset by the present study to demonstrate the effect of the multi-feature fusion strategy. The consistent results of the subjective and objective assessments of these ablation experiments are demonstrated in Table 5.

**Table 5:** Performance of ablation experiments on the SAUD dataset.

Features	SROCC	KROCC	PLCC
$f_1$	0.4503	0.3146	0.4753
$f_2$	0.5155	0.3631	0.5595
$f_3$	0.5634	0.3998	0.6096
$f_4$	0.5339	0.3784	0.5802
$f_5$	0.4903	0.3495	0.5208
$f_6$	0.6147	0.4456	0.6442
L-features	0.7080	0.5238	0.7271
C-features	0.6468	0.4706	0.6788
Overall	**0.8354**	**0.6467**	**0.8401**

The best values are in bold.

As indicated in Table 5, it is observed that the increasing feature numbers utilized leads to improved performance compared to using a single feature, particularly in the case of the methods utilizing only luminance or chromatic features. Specifically, the values of the three evaluation metrics, namely SROCC, KROCC, and PLCC, of the method utilizing luminance features are 0.7080, 0.5238, and 0.7271 respectively, representing a performance improvement of approximately 15$\%$ when compared with the best performing single feature method, with improvements of 0.0933, 0.0782, and 0.0829 respectively. With an increase in the number of features utilized, the proposed method demonstrates improved performance across the assessment metrics of SROCC, KROCC, and PLCC, with values reaching 0.8354, 0.6467, and 0.8401 respectively. These performance gains are particularly evident when compared against an method utilizing only luminance and chromatic features, with corresponding SROCC improvements of 0.1274 and 0.1886, increases in KROCC of 0.1229 and 0.1761, additionally, significant increases in PLCC values of 0.113 and 0.1613 respectively. The analysis conducted in this paper indicates that through the fusion of luminance and color component features, the proposed NMFC method achieves optimal performance across all assessment metrics, with the highest degree of both subjective and objective agreement. The experimental results illustrate the performance of individual feature. Meanwhile, the results demonstrate the effective of multi-feature fusion in UIQA.

**Table 6:** Performance comparisons of the proposed method on natural image datasets and screen content image datasets.

	Method	LIVE	TID2013	SIQAD	LIVEMD
SROCC	SSIM	0.9269	0.6370	**0.7521**	0.6786
	PSNR	0.8756	0.5531	0.5608	0.6771
	FSIM	0.9610	**0.8015**	0.5819	0.6835
	VIF	**0.9639**	0.6679	0.6314	0.8823
	SSEQ	0.9033	0.6162	0.7287	0.8667
	BIQI	0.8796	0.4939	0.7320	**0.8905**
	NMFC	0.9369	0.6735	0.7458	0.8742
KROCC	SSIM	0.7526	0.4636	0.5543	0.5006
	PSNR	0.6865	0.4027	0.4226	0.5003
	FSIM	**0.8379**	**0.6289**	0.4250	0.6727
	VIF	0.8282	0.5147	0.4577	0.6969
	SSEQ	0.7364	0.4490	0.5393	0.6832
	BIQI	0.7312	0.3493	0.5436	**0.7174**
	NMFC	0.7957	0.4940	**0.5559**	0.6855
PLCC	SSIM	0.9243	0.6524	0.7461	0.6198
	PSNR	0.8723	0.5734	0.5508	0.7716
	FSIM	0.9597	**0.8589**	0.5902	0.8359
	VIF	**0.9604**	0.7720	0.7066	**0.9144**
	SSEQ	0.9017	0.6943	0.7704	0.8805
	BIQI	0.8823	0.5935	0.7740	0.9052
	NMFC	0.9404	0.7191	**0.7830**	0.8829

### Sensitivity to image type

In order to verify the generalizability of the method in this paper, we conduct comparison experiments on singly distorted synthetic datasets LIVE, TID2013, screen content datasets SIQAD,multiply distorted synthetic datasets LIVEMD datasets, and compare the performance of the NMFC method proposed in this paper with existing classical IQA methods such as SSIM^46^, PSNR^47^, FSIM^48^, VIF^49^, etc.

The overall assessment results of the proposed method and other six classical image quality methods on three datasets are given in Table 6, and the better two results are bolded in the table. According to the results in Table 6, it can be seen that the method of this paper achieves better results on all three datasets. Firstly, on the SIQAD dataset, the proposed method outperforms other image quality evaluation methods, and the performance of SROCC and PLCC is almost higher than 0.2 compared to the worst method; still have some competitiveness on LIVE dataset, SROCC, KROCC, and PLCC values are only below FSIM and VIF methods, but the difference is not large, PLCC is only 0.027, 0.0422 and 0.02 lower than the optimal assessment results on three assessment metrics. On the TID2013 dataset, the performance of this method is only worse than FSIM method and achieves sub-optimal results, but the method in this paper is a no-reference method, compared with the FSIM method, which requires reference information, the proposed method has a wider applicable scope. Meanwhile, based on the results presented in the table, it can be observed that the evaluation performance of FSIM and VIF methods surpasses that of the proposed method on certain datasets, however, it is noted that their performance on the SIQAD dataset is suboptimal, leading to a significant performance gap with the proposed method. Finally,in the evaluation on the LIVEMD dataset, our proposed method demonstrates slightly lower performance compared to the VIF and BIQI methods. VIF is a full reference algorithm that relies on more reference information, while our proposed method operates without any reference information and still achieves comparable results to VIF. The maximum difference in each evaluation index between our method and VIF is less than 0.035. Additionally, the SROCC, KROCC, and PLCC values of our method are 0.0163, 0.0319, and 0.0196 lower than the no-reference method BIQI. However, considering that our proposed method is specifically designed for underwater images, a certain gap in performance is still acceptable.

**Figure 9:**
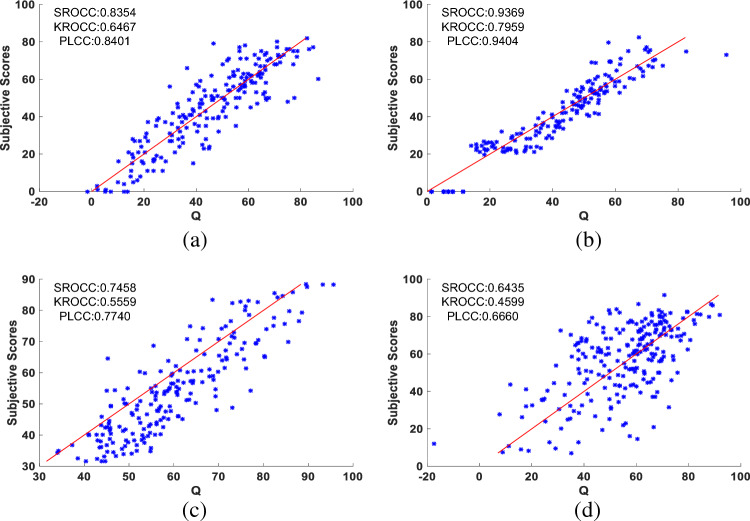
Scatter plots of the predicted quality index Q versus Subjective Scores for the test set. The x-axis is the predicted quality index Q and the y-axis is the subjective value. The red line represents the ideal linear correlated line. (**a**) SAUD dataset. (**b**) LIVE dataset. (**c**) SIQAD dataset. (**d**) LIVEC dataset.

To demonstrate the generalizability of the proposed method, scatter plots were utilized to illustrate the correlation between the quality prediction results and subjective prediction results of the proposed method across various image categories, including underwater images, synthetic distortion images, screen content images, and real images in the wild. Fig. 9 displays the scatter plot depicting the predicted scores versus the subjective scores of the proposed method on SAUD, LIVE, SIQAD, and LIVEC datasets. From the a–c in Fig. 9, it can be observed that the quality prediction results of the proposed method align closely with the subjective evaluation results for underwater images, synthetic distortion images, and screen content images, where most samples compactly gather around the linear correlated line. It demonstrates that the proposed method predictions are highly consistent with human subjective perception. This observation provides evidence that the proposed method is capable of adapting to the task of image quality assessment in these scenarios. However, when evaluating the proposed method on the real scene dataset LIVEC, the SROCC, KROCC, and PLCC values are only 0.6435, 0.4599, and 0.6660, respectively, indicating not performing well. Notably, Fig.9d clearly illustrates that the points demonstrate a relative degree of dispersion and do not exhibit a tightly clustered distribution around the linear correlated line. This highlights a substantial disparity between subjective and objective consistency. Therefore, further improvements are necessary to enhance the performance of the proposed method for image quality assessment in authentic settings.

In summary, it is evident that the proposed method is resilient and can basically adapt to various distortion types and changes in image characteristics across different scenarios, demonstrating a degree of generalizability.

### Stability analysis

**Figure 10:**
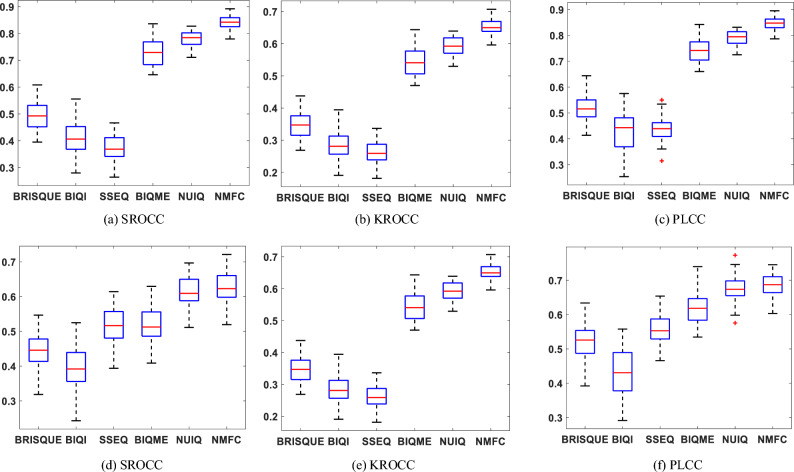
Box plot of IQA methods on SAUD and UIED. (**a–c**) SAUD database. (**d–f**) UIED database.

This paper conduct performance comparison on SAUD and UIED datasets to verify the stability of the proposed method NMFC in this paper. Fig. 10 gives the box plots of the assessment results corresponding to this method and different IQA methods on SAUD and UIED datasets. The horizontal axis of the box plots represents the various IQA methods, while the vertical coordinates depict the objective assessment scores obtained through each method for the given set of images. The shape of each box corresponding to each method describes the distribution of the data and reflects the assessment effects of the different IQA methods. As shown in Fig. 10, our findings on both the SAUD and UIED datasets indicate that, in comparison with other IQA techniques, the boxes corresponding to the proposed method on each assessment index are smaller. A smaller box size indicates less fluctuation in the method’s assessment of image quality. Moreover, the distance between the upper and lower edges of the boxes corresponding to this method is small, and there are no outliers, which demonstrates that the proposed method is more stable in assessing the quality of underwater images, compared to other IQA methods. Additionally, the median score for our proposed method is higher relative to other methods, indicating better assessment accuracy. Overall, our proposed method exhibits high stability and is effective in assessing the image quality of underwater images.

## Conclusion

In this paper, an UIQA method based on color space multi-feature fusion is proposed. The proposed method extract morphological features, histogram features, moment statistics, and other features from the color space-based transformed image and perform multi-feature fusion to assess the quality of underwater images. Experimental results demonstrate that our proposed method achieves high accuracy and robustness for UIQA, and is consistent with human subjective perception. Additionally, our method can satisfy the demands of both natural image and screenshot IQA .

The existing IQA methods proposed in this paper rely on artificial features and overlook the impact of complex real distortions on image quality in real scenes, posing a challenge for evaluating image quality in realistic environments. Enhancing the performance of the method constitutes the primary objective for future research endeavors. To address this issue, our focus will be on semantically mining the feature maps proposed in this paper, combined with deep neural networks, aiming to achieve a more efficient and effective approach to image quality assessment.

## Data Availability

Data supporting the results of this study are available from the corresponding author upon reasonable request, including source code and experimental results.
